# Impact of palladium nanoparticles on plant and its fungal pathogen. A case study: *Brassica napus–Plenodomus lingam*

**DOI:** 10.1093/aobpla/plad004

**Published:** 2023-02-02

**Authors:** Lukáš Maryška, Barbora Jindřichová, Jakub Siegel, Kamil Záruba, Lenka Burketová

**Affiliations:** Institute of Experimental Botany, The Czech Academy of Sciences, Rozvojová 313, 165 02, Prague 6 – Lysolaje, Czech Republic; University of Chemistry and Technology Prague, Technická 5, 166 28, Prague 6 – Dejvice, Czech Republic; Institute of Experimental Botany, The Czech Academy of Sciences, Rozvojová 313, 165 02, Prague 6 – Lysolaje, Czech Republic; University of Chemistry and Technology Prague, Technická 5, 166 28, Prague 6 – Dejvice, Czech Republic; University of Chemistry and Technology Prague, Technická 5, 166 28, Prague 6 – Dejvice, Czech Republic; Institute of Experimental Botany, The Czech Academy of Sciences, Rozvojová 313, 165 02, Prague 6 – Lysolaje, Czech Republic

**Keywords:** Palladium, nanoparticle, plant defence, *Brassica napus*, *Leptosphaeria maculans*, *Plenodomus lingam*

## Abstract

The technological exploitation of palladium or palladium nanoparticles (PdNPs) is increasing, and their wider usage relates to an unwanted release of pollutants into the environment, raising public health concerns about the infiltration of palladium into the consumption chain. This study focuses on the effect of spherical gold-cored PdNPs of 50 ± 10 nm diameter stabilized by sodium citrate on the interaction between an oilseed rape (*Brassica napus*) and the fungal pathogen *Plenodomus lingam.* Pretreatment of *B. napus* cotyledons with PdNPs suspension 24 h before but not 24 h after inoculation with *P. lingam* resulted in a decrease in the extent of disease symptoms; however, this effect was caused by Pd^2+^ ions (35 mg l^–1^ or 70 mg l^–1^). Tests to determine any direct antifungal activity on *P. lingam in vitro* demonstrated that the residual Pd^2+^ ions present in the PdNP suspension were responsible for the antifungal activity and that PdNPs themselves do not contribute to this effect. *Brassica napus* plants did not show any symptoms of palladium toxicity in any form. PdNPs/Pd^2+^ slightly increased the chlorophyll content and the transcription of *pathogenesis-related gene 1 (PR1),* indicating the activation of the plant defence system. We conclude that the only toxic effect of the PdNP suspension was on *P. lingam* via ions and that PdNPs/Pd^2+^ did not have any deleterious effect on the *B. napus* plants.

## Teaser

Palladium nanoparticles (PdNPs) are released into nature as pollutants mainly through the operation of motor vehicles or industrial production and have the potential to enter the food chain. Their effects on plants were studied in a pathosystem of oilseed rape and its fungal pathogen *Plenodomus lingam*. Palladium showed both an antimicrobial effect and an activation of the plant defence system, but these effects were found in palladium ions and not in nanoparticles, which showed no toxicity to the plant or the fungus. On the other hand, the results indicate a potential hazard from PdNPs—released ions—that may occur in a natural environment.

## Introduction

Nanoparticles (NPs) are defined as particles ranging in size from 10 to 1000 nm. They can be found naturally, for example as organic units (proteins, polysaccharides, viruses, etc.) or as inorganic compounds (metal oxides, metals, aluminosilicates, etc.). NPs are also produced artificially in large quantities, e.g. as sunscreens, in antimicrobial compounds, in pollution from factories, as electronic waste, etc., and are now released into the environment to a larger extent than previously. Due to their solubility and stability, NPs can travel in water or air for long distances (Rinkovec *et al.*, 2018). NPs enter the environment, where they can accumulate in plants and join the consumption chain. Thus, their possible impact on living organisms as contaminants is widely discussed (Burketova *et al.*, 2022).

After decades of research, a large number and variety of synthesis types and NPs are available. The most common are spheres, rods, and triangles, but cubes, cages, stars, and other variations can also be prepared. The characterization of NPs is dependent on the material chosen and the matrix in which the NPs are synthesized. The selection of matrix materials influences the inherent properties of NPs, such as aqueous solubility or stability, surface charge, permeability, degree of biodegradability, biocompatibility, and toxicity (Mohanraj and Chen, 2006).

Various physical and chemical parameters mean different NP antimicrobial activities. For many of them, smaller NPs and/or a specific surface charge, caused by stabilization reagents, have higher toxic effects (Martinez-Gutierrez *et al.*, 2010; Kim *et al.*, 2013; Mukha *et al.*, 2013; Elmehbad and Mohamed, 2020). The exact mechanisms of the antimicrobial effects of NPs are still unknown, but a few hypotheses exist. Li *et al.* (2008) proposed a possible antibacterial mechanism of silver NPs. First, NPs adhere to a surface of the bacterial cell wall. Larger NPs will stay on the surface and smaller NPs will penetrate directly into the cell, where they can cause mechanical destruction of DNA. In both cases, the main toxic effect is probably caused by the release of silver ions from the NPs. Ions have a destabilizing potential on the membrane, causing proton leakage. This can interact with thiol-containing proteins or phosphates of DNA in the cell and affect their functions (Sanchez-Lopez *et al.*, 2020). The antibacterial effect of NPs is dependent on the type of bacteria. Gram-negative bacteria are covered by a thin layer of lipopolysaccharides and peptidoglycans, whereas Gram-positive bacteria have a thicker layer of peptidoglycans (Slavin *et al.*, 2017). Many studies have found that Gram-positive bacteria, due to their covering, are more resistant to the toxic effect of NPs (Cavassin *et al.*, 2015; Dorobantu *et al.*, 2015; Feng *et al.*, 2000).

Under field conditions, we still cannot find common applications for the regulation of plant diseases, but smartly designed NPs have the potential to be used for the stimulation of agricultural crop production, since they can be used as plant growth stimulators, nanofertilizers, or soil improving agents. The positive effects of metallic NPs on plants have been shown for silver and metal oxides (Goswami *et al.*, 2019; Landa, 2021). On the other hand, higher concentrations of metallic NPs can cause wider damage to plants. The phytotoxicity of silver NPs was studied on cucumber and lettuce. In the presence of silver NPs, seed germination is reduced for cucumber seeds, but in the case of lettuce, a germination index is better comparable with control seeds (Barrena *et al.*, 2009). Another study showed the impact of silver NPs and Ag^+^ on the root elongation of barley, lettuce, and radish, cultivated under hydroponic conditions (Gruyer *et al.*, 2014). They showed that there is a positive response of root elongation in barley to lower concentrations of silver NPs, but a reduction in root length at higher concentrations. A reduction in root length for lettuce was observed in each chosen concentration of silver NPs and for radish, there was no significant effect of silver NPs. In the plants *Oryza sativa*, *Triticum aestivum*, and *Vicia faba*, silver causes other damage, such as the destruction of cell walls, a genotoxic effect, and oxidative bursts (Mazumdar and Ahmed, 2011; Patlolla *et al.*, 2012; Rastogi *et al.*, 2017). Another metal that affects plant growth is gold. Results showed that gold NPs reduce the elongation of the primary root and reduce the length and number of lateral roots under *in vitro* conditions in *A. thaliana* plants. Few of the metal NPs can be used for the management of plant disease. The most explored metal is silver. The inhibition effects of silver NPs against the fungal agents of powdery mildew have been found in cucumber and pumpkin. In field experiments, silver NPs of 50 and 100 ppm had significant effects on these pathogens (Lamsal *et al.*, 2011). Other numerous effects of silver or gold NPs on plants and microorganisms have been also reported (for detailed information see Kaveh 2013; Macurkova *et al.*, 2021; Yan and Chen, 2019; Burketova *et al.*, 2022).

Another element from the noble metal group is palladium, which belongs to the platinum group elements (PGEs). In comparison to other non-essential toxic elements such as mercury, lead, or cadmium, the concentration of palladium in the environment is relatively low. The main source of palladium is traffic, where the palladium content is relatively high, especially in road dust or roadside soils (Wiseman *et al.*, 2016; Leopold *et al.*, 2017). In the most contaminated soil samples, the concentration of palladium ranges from tenths to thousands of nanogram per gram (Aarzoo *et al.*, 2022). Palladium’s solubility and great mobility make it the most toxic element of the PGEs (Ek *et al.*, 2004; Havelkova *et al.*, 2014). The cultivation of *Sinapis alba* plants in the presence of palladium nanoparticles (PdNPs) in soil or hydropony caused a slight colorization of the leaves with darker green along the veins. Roots in the presence of palladium nitrate had a yellow-brown color, and those cultivated with PdNPs were dark gray. The authors suggested that this effects could be due the accumulation of palladium on root surface (Kinska *et al.*, 2018). PdNPs have also antimicrobial activity. Antibacterial and antifungal activity of synthetized NPs have been recently reported by Bi and Ahmad (2022) and Osonga *et al.* (2020). They showed that synthetized PdNPs are represing bacterial growth of *Staphylococcus auereus*, *Escherichia coli*, and *Pseudomonas aeruginosa* and supressing a fungal growth of *Colletotrichum gloeosporioides* and *Fusarium oxysporum*.

The environment is slowly adapting to nanoparticle pollution. Our study focuses on the effects of gold-cored PdNPs and Pd^2+^ ions on plant physiological parameters and immunity, which could affect plant performance in field. We chose an economically important crop, oilseed rape, and its important fungal pathogen *Plenodomus lingam* as an experimental model. In addition to the effects of palladium on plants, a direct antimicrobial activity of PdNPs and Pd^2+^ ions was measured.

## Material and Methods

### Preparation of NPs

PdNPs have been synthesized with a slightly modified seed-mediated approach developed by Chen *et al.* (2010). Hydrochloric acid (72.8 µL, 35 %, w/w) was added to aqueous potassium palladate salt solution (2 mM, 207 mL) to generate H_2_PdCl_4_. Subsequently, 20.5 mL of sodium citrate (1 %, w/w), 8.2 mL of hydrogen peroxide (30 %, w/w), and 20.5 mL of gold seeds were added. The reaction mixture was stirred at room temperature for 1.5 h. Before the experiment, the NPs were purified by removing unreacted chemicals, especially Pd^2+^ ions. The PdNP solution was placed on a TLA 100.3 rotor and centrifuged at 541 000*g* on an Optima MAX-XP ultracentrifuge (Backman Coulter, IN, USA) for 30 minutes. After this, the supernatant was removed and the concentrate was diluted to the original volume by pure buffer (2.0 mM sodium citrate). Transmission electron microscopy (TEM) images were measured using JEOL JEM-1010 (JEOL Ltd., Japan) operated at 400 kV. The particle size was measured from the TEM micrographs and calculated taking into account at least 500 particles.

Gold seeds for the preparation of PdNPs were freshly prepared prior to each PdNP batch and synthesized according to Siegel *et al.* (2018). The concentration of Pd in the PdNPs solution was determined by atomic absorption spectroscopy on a VarianAA880 device (Varian Inc., Palo Alto, CA, USA) using a flame atomizer at a wavelength of 242.8 nm. The typical uncertainty of concentration determined by this method was less than 3 %. Ultraviolet–visible spectroscopy (UV–vis) was used to study the optical properties of colloidal dispersions of PdNPs. Absorption spectra were recorded on a Lambda 25 spectrophotometer (PerkinElmer Inc., Waltham, MA, USA) in the spectral range 300–800 nm with a 1-nm data step, scan speed of 240 nm min^–1^. Measurements were accomplished in a polystyrene cuvette with a 1-cm light path.

### Plant and pathogen cultivation

Plants of *B. napus* cv. Columbus were grown hydroponically in perlite with Steiner’s (Steiner, 1984) cultivation medium under defined conditions (day/night: 14/10 h, 24/20 °C, photon flux density 150 μmol m^–2^ s^–1^). Cotyledons were used for all experiments.

The fungus *P. lingam* (anamorph *Phoma lingam*, Balesdent *et al.* [2001], isolate JN2) was cultivated on V8 solidified medium (20 % V8 vegetable juice, Campbell soup company, Camden, NJ, USA, 3 g l^–1^ CaCO_3_, 15 g l^–1^ agar). Sporulation cultures and conidia suspension were prepared according to Sasek *et al.* (2012a). After harvesting, the spores were diluted to 10^8^ spore mL^–1^ and stored at ^–^20°C for a maximum of 6 months.

### *In vitro* antifungal assay

The antifungal activity of PdNPs and Pd^2+^ ions was measured according to the method previously described by Jindrichova *et al.* (2014). Briefly, spores of *P. lingam* JN2 with constitutive expression of GFP (JN2::GFP) (Sasek *et al.*, 2012a) were suspended into 5 × 10^4^ spore mL^–1^ in a Gamborg B5 medium (Duchefa Biochemie BV, Haarlem, Netherlands), supplemented with 0.3 % saccharose and 10 mM MES (pH 6.8). Fifty μl of conidia suspension was pipetted into a black 96-well plate and 50 µL of palladium suspension/solution was then added. The final concentrations of PdNPs used were 10, 20, 40, and 80 mg l^–1^, and the final concentrations of K_2_PdCl_4_ (source of Pd^2+^ ions used for PdNPs synthesis) were 0.35, 0.65, 1.25, and 2.5 mg l^–1^, which corresponded with the concentrations of residual Pd^2+^ ions present in PdNPs suspensions quantified by the inductively coupled plasma mass spectrometry. Both PdNPs and K_2_PdCl_4_ were suspended/dissolved in 2 mM sodium citrate. As a control treatment, 2 mM sodium citrate was used. The covered and parafilm-sealed plates were cultivated at 26 °C and in the dark. Relative fluorescence was measured using Infinite F200 plate reader TECAN (Tecan, Männedorf, Switzerland) with 485/20 nm filters for excitation and 535/25 nm for emission every 24 h for 5 days. The fungistatic activity of PdNPs and Pd^2+^ ions was measured using the modified method described above. After 72 h’ incubation of 50 μL of conidia suspension, 50 μL of tested solution was pipetted into the well. Relative fluorescence was measured every 24 h for 4 days after adding Pd suspension.

### Plant treatment

Cotyledons of 13- or 15-day-old plants were used for PdNPs, Pd^2+^ ions and Pd mixture (Pd mix) treatment. The Pd mix contained both PdNPs and K_2_PdCl_4_ (Pd^2+^ ions) in the ratio of 1:1. All Pd suspensions/solution (PdNPs, K_2_PdCl_4_ [Pd^2+^ ions], and a Pd mix) were suspended/disolved in 2 mM sodium citrate. The final concentrations of Pd were 17, 35 and 70 mg l^–1^ for the inoculation test, and the 35 mg l^–1^ (PdNPs, Pd^2+^ ions) and 70 mg l^–1^ (Pd mix) for hydrogen peroxide detection, the measurement of antioxidant enzymes activity, measurement of plant fitness, determination of chlorophylls and carotenoids, and gene analysis. Sodium citrate of a 2-mM concentration was used as the control treatment (mock). Cotyledons were treated by infiltration using a syringe without a needle until full leaf saturation.

### Inoculation test

Cotyledons of 14-day-old plants were inoculated by the infiltration of a conidia suspension of *P. lingam* JN2 (10^5^ spore mL^–1^) using a needleless syringe until complete leaf saturation. Infected leaves were scanned 10–12 days after inoculation. *Plenodomus Lingam* lesions were evaluated by image analysis using APS Asses 2.0 software (American Phytopathological Society, St. Paul, MN, USA). The relative area of the lesions was referred to mock plants when the value for mock plants was set at 100 % with 24 cotyledons of each treatment used for analysis.

### Hydrogen peroxide detection

Hydrogen peroxide was detected using 3,3’-diaminobenzidine (DAB) by the method of Thordal-Christensen *et al.* (1997). The cotyledons were cut and infiltrated by vacuum with 1 mg mL^–1^ of DAB solution in 10 mM Tris buffer, pH 7.8. The leaves were incubated for 4 h in darkness at room temperature. After incubation, chlorophyll was extracted from leaves by several changes of ethanol. DAB forms a reddish-brown polymerization product in the presence of H_2_O_2_ and peroxidase (PX).

### Activity of antioxidant enzymes

Plant extracts were prepared by the homogenization of 1 g of fresh *B. napus* cotyledons in an ice-cold mortar with 6 mL of 50 mM Tris buffer, pH 7.8, containing 1 mM EDTA and 7.5 % polyvinylpolypyrrolidone. Homogenates were centrifuged at 19 000*g* for 20 minutes at 4°C. Supernatants were used for enzyme activity assays.

The activity of catalase (CAT, EC 1.11.1.6) was measured according to Aebi (1984), the method based on the decrease of absorbance at 240 nm. The reaction mixture contained 0.1 M Na-phosphate buffer, pH 6.8, 1 mM EDTA, 269 mM H_2_O_2_, and 50 μL of the enzyme extract in the final volume of 2 mL. The concentration of H_2_O_2_ was calculated using the extinction coefficient of H_2_O_2_ (0.04 mM^–1^ cm^–1^). CAT activity was expressed as the decrease in H_2_O_2_ (μM min^–1^ gFW^–1^).

The activity of guaiacol peroxidase (GPX, EC 1.11.1.7) was determined according to Chance and Maehly (1955). The reaction mixture contained 0.1 M Na-phosphate buffer, pH 6.8, 1 mM EDTA, 30 mM H_2_O_2_, 50 mM guaiacol, and 50 μL of enzyme extract in the final volume of 1 mL. The formation of tetraguaiacol was detected at 480 nm. The concentration of tetraguaiacol was calculated using the extinction coefficient of tetraguaiacol (26.6 mM^–1^ cm^–1^). GPX activity was expressed as the amount of tetraguaiacol formed (μM min^–1^ g FW^–1^).

The activity of ascorbate peroxidase (APX, EC 1.11.1.11) was estimated according to Nakano and Asada (1981) by monitoring the ascorbate oxidation at 290 nm. The reaction mixture contained 0.2 M Tris buffer, pH 7.6, 3 mM EDTA, 3.75 mM H_2_O_2_, 5.625 mM ascorbate, and 100 μL of enzyme extract in the final volume of 2 mL. The concentration of ascorbic acid was calculated using the extinction coefficient of ascorbic acid (2.8 mM^–1^ cm^–1^). APX activity was expressed as the decrease in ascorbic acid (μM min^–1^ gFW^–1^).

The activity of glutathione reductase (GR, EC 1.8.1.7) was measured according to Klapheck *et al.* (1990) as consumption of NADPH at 340 nm. The reaction mixture contained 0.2 M Tris buffer, pH 7.6, 3 mM EDTA, 3.75 mM NADPH, 4.16 mM glutathione disulfide (GSSG), and 50 μL of enzyme extract in the final volume of 1 mL. The concentration of NADPH was calculated using the extinction coefficient of NADPH (6.2 mM^–1^ cm^–1^). GR activity was expressed as the amount of NADPH (μM min^–1^ gFW^–1^).

### Evaluation of plant growth

The cotyledon area and the dry plant matter were measured 11 days after the infiltration of Pd suspension/solution. For the cotyledon area, 12 cotyledons from 12 plants per treatment were scanned and their area was evaluated by APS Assess 2.0 image analysis software (American Phytopathological Society, St. Paul, MN, USA) and compared to the mock plants (sodium citrate treatment). For dry matter evaluation, 12 cotyledons of 12 plants per treatment were dried at 100°C to a constant weight. The proportion of dry matter was calculated as the ratio of dry weight to fresh weight.

### Determination of the concentration of chlorophylls and carotenoids

The concentration of chlorophylls *a* and *b* and of the carotenoids was determined (Porra *et al.*, 1989) in cotyledons 11 days after Pd suspension/solution treatment. The chlorophylls and carotenoids were extracted from 6 leaf discs (6 mm diameter disc per cotyledon of 6 plants) in 3 mL of *N*,*N*’-dimethylformamide incubated overnight in the dark until decolorization. Final absorbance was measured at 480, 646.8, 663.8 and 710 nm using a spectrophotometer (Helios β, Unicam, UK). The contents of the chlorophylls (Porra *et al.*, 1989) and carotenoids (Wellburn, 1994) were expressed as mg l^–1^ mm^–2^ of leaf area.

### Gene transcription analysis

Gene transcription was evaluated using qPCR in cotyledons 24 hours after treatment. RNA was isolated from treated *B. napus* cotyledons 24 hours after infiltration. The weight of the collected samples was around 150 mg (10–12 discs with a radius of 6 mm). Isolation and purification were performed with the Spectrum^TM^ Plant Total RNA Kit commercial kit (Sigma-Aldrich, St. Louis, MO, USA). Every step of isolation was performed according to the protocol. The quality and concentration of RNA was evaluated on NanoDrop 1000 (Thermo Scientific, Waltham, MA, USA). One μg of RNA was treated with the DNAfree^TM^ kit (Ambion, Austin, TX, USA) and converted to cDNA by reverse transcription of M-MLV RNase H-point mutant (Promega Corp., USA) and anchored oligo dT21 primer (Metabion, Martinsried, Germany). The qPCR reaction contained the equivalent of 6.25 ng of RNA in LightCycler^®^ 480 SYBR Green I Master (Roche, Basel, Switzerland). The final volume of reaction was 10 µL and was performed in a 96-well plate using LightCycler^®^ 480 (Roche, Basel, Switzerland). The PCR conditions were 95°C for 10 minutes followed by 45 cycles of 95°C for 10 seconds, 55°C for 20 seconds, and 72°C for 20 seconds, followed by a melting curve analysis. Threshold cycles and melting curves were calculated using LightCycler^®^480 software (Roche, Basel, Switzerland). The level of relative transcription was calculated with an efficiency correction and normalized to the reference gene *Actin*. A list of primers is shown in Table S1.

### Statistical analysis

The experiments were carried out in three independent biological replicates (i.e. three separate experiments not conducted in parallel at the same time). Data from individual treatments of all replicates were averaged and analyzed using one-way ANOVA after a Tukey honestly significant difference multiple mean comparison post hoc test, *P* < 0.05.

## Results

### Characterization of PdNPs

**Figure 1.**
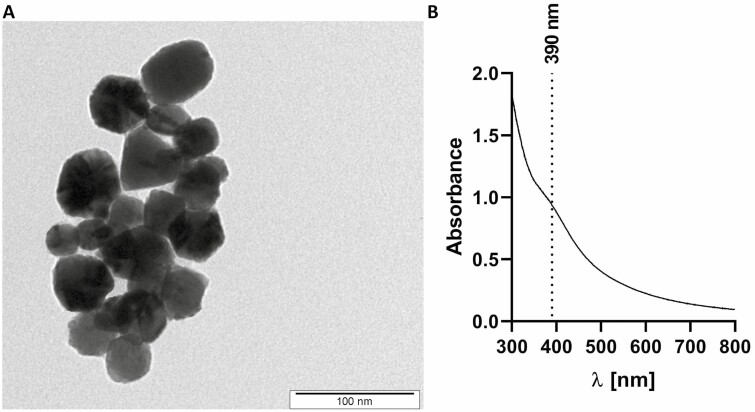
TEM image of prepared and purified PdNPs (A) together with corresponding absorption spectrum (B).

The size distribution of the prepared and purified PdNPs was evaluated from TEM images (Fig. 1A). They were approximately spherical in shape, with a diameter of 50 ± 10 nm. The absorption spectrum of the corresponding double diluted sample of PdNP can be seen in Fig. 1B, with plasmonic absorption at about 390 nm.

### The antifungal effect of PdNPs and Pd^2+^ ions on *P. lingam in vitro*

**Figure 2.**
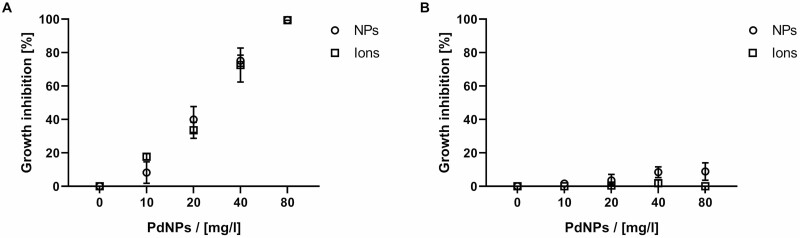
Antifungal activity of PdNPs and Pd^2+^ ions against *P. lingam in vitro* (spore germination (A) and mycelium growth (B)). PdNPs were used at concentrations ranging from 10 to 80 mg l^–1^ and the Pd^2+^ ions tested ranged in concentrations from 0.35 to 2.5 mg l^–1^; these quantities corresponded to remaining Pd^2+^ ions in the suspension of PdNPs. The data represent mean values of three independent experiments. Error bars represent mean ± SE values from three independent experiments. ○ NPs ◻ Pd^2+^ ions.

During the purification of NPs, the majority of the unreacted ions was removed; however, the final suspension of NPs still contains a residual concentration of ions. These ions can cause false positive results. Consequently, the antimicrobial effect of residual Pd^2+^ ions on *P. lingam* was tested. *In vitro* studies of the antifungal effects of the Pd suspension/solution have been focused on the impact of Pd on *P. lingam* spore germination and the impact on mycelium growth (Fig. 2). PdNP concentrations used ranged in concentrations from 10 to 80 mg l^–1^. The tested concentrations of Pd^2+^ ions ranged from 0.35 to 2.5 mg l^–1^; these concentrations corresponded to the remaining concentration of Pd^2+^ ions in PdNPs suspension. The determined IC_50_ of Pd^2+^ ions inhibiting the germination of the *P. lingam* spores was 1.06 mg l^–1^. Figure 2A shows an inhibition effect of PdNPs and Pd^2+^ ions, but the level of inhibition was the same in PdNP treatment as in Pd^2+^ ion treatment. The germination of spores in NP suspension and Pd^2+^ions did not differ statistically, suggesting that the toxic effect of PdNPs was caused by residual amounts of Pd^2+^ ions.

The inhibition of *P. lingam* mycelium growth was also monitored. PdNP suspension at a concentration of 10–80 mg l^–1^ and Pd^2+^ solution at concentrations of 0.35–2.5 mg l^–1^ were added to the grown mycelium of *P. lingam* 72 hours after germination. There was no significant difference between PdNPs, Pd^2+^ and mock control (Fig. 2B). Pd^2+^ ions had an inhibitory effect exclusively on spore germination, but they had no effect on mycelium.

### The influence of PdNPs and Pd^2+^ ions on the development of *P. lingam* symptoms in *B. napus* plants

Metal ions have been shown previously to impact plant immunity (Morkunas *et al.*, 2018). For this reason, we investigated the effects of Pd on *B. napus* resistance to *P. lingam*, both in cotyledons pre-treated with a Pd suspension/solution 24 h before inoculation and 24 h after inoculation with *P. lingam*.

Since concentration of palladium in nature exceeds 50 mg kg^–1^ in the most contaminated soils (Wiseman *et al.*, 2016), the concentrations of total Pd of 17, 35 and 70 mg l^–1^ were chosen in the experiments. The treatment of plants by the residual concentration of Pd^2+^ ions detected in PdNPs suspension had no impact on the lesion area’s development (data not shown). Prepared PdNPs in 2.0 mM sodium citrate were stable within one months at room tempereature (data not shown). On the other hand, metal NPs can release metal ions in a natural environment (Gioria *et al.*, 2020). For this reason, Pd^2+^ ions and PdNPs were used at similar concentrations. Pd^2+^ ions and a Pd mix were used to study potential additional effect of PdNPs to the Pd^2+^ ions. The Pd mix contained PdNPs and Pd^2+^ ions in a ratio of 1:1 (NPs:ions), which represented the same total Pd concentration as in PdNPs suspension or Pd^2+^ solution.

**Figure 3.**
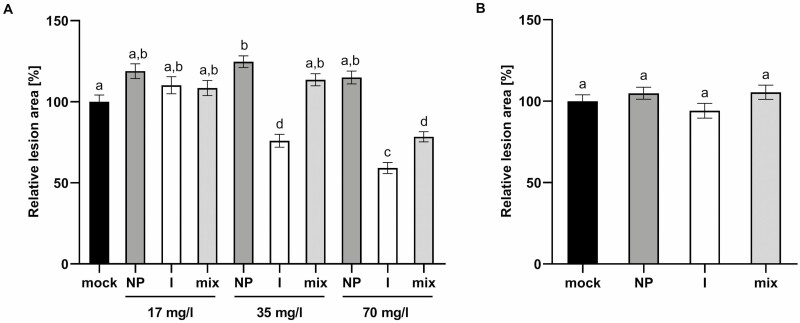
Effect of PdNPs and Pd^2+^ ions on the development of *P. lingam* symptoms in *B. napus* cotyledons. *B. napus* plants were treated with PdNPs (NP), Pd^2+^ ions (I), and Pd mix (mix) with a concentration of 17, 35, or 70 mg l^–1^ 24 hours before inoculation (A) or treated with 35 mg l^–1^ PdNPs, Pd^2+^ ions and 70 mg l^–1^ Pd mix 24 hours after inoculation with *P. lingam* (B). Pd mix contained PdNPs and Pd^2+^ at a 1:1 ratio (NPs:ions). Disease symptoms were evaluated as percentage of the lesion area to the leaf area 11 days after inoculation. Data represent means ± SE values, *n* = 72, *P* < 0.05.

Except for the treatment with 35 mg l^–1^ PdNPs, none of the selected PdNPs concentrations significantly increased *P. lingam* lesions in Pd pre-treated plants (Fig. 3A). Pre-treatment by 35 mg mL^–1^ PdNPs increased the lesion area by 20 % compared to the mock treatment. The pre-treatment by Pd^2+^ ions caused the reduction of *P. lingam* symptom development. In the case of Pd^2+^ ion pre-treatment with concentrations of 35 and 70 mg l^–1^, the lesions were significantly decreased compared to the mock treatment. The Pd^2+^ ions at a concentration of 35 mg l^–1^ reduced the area of the lesion by 20 %, and a concentration of 70 mg l^–1^ by 40 %. Only the lowest concentration of Pd^2+^ ions (17 mg l^–1^) had no effect. The plant pre-treatment by the Pd mix mimicked the effect of Pd^2+^ ions on the development of the lesion area. Concentrations of a total Pd of 17 and 35 mg l^–1^ had no effect on *P. lingam* symptoms. Only pre-treatment of a total 70 mg l^–1^ Pd in the mix decreased the lesion area by 20 %, the same impact as treatment with a Pd^2+^ ion concentration of 35 mg l^–1^. The results show that the reduction of *P. lingam* lesions was caused by the presence of Pd^2+^ ions.

In experiments on post-inoculation Pd treatment (Fig. 3B), only Pd concentrations efficient in pre-treatment were used, i.e. 35 mg l^–1^ for Pd in NP form and ion form and of 70 mg l^–1^ in Pd mix. Post-inoculation treatment. with PdNPs, Pd^2+^ ions and a Pd mix were tested by the infiltration of suspensions into cotyledons of *B. napus* 24 hours after their inoculation with *P. lingam* spores. Post-treatment had no impact on the lesion development of *P. lingam* in *B. napus* cotyledons (Fig. 3B).

### Activation of plant defense pathways by PdNPs and Pd^2+^ ions

Numerous metal ions activate defence mechanisms in plants such as MAPK signalling, ROS, microRNAs, phytohormones and others (Jalmi *et al.*, 2018). Since our above results indicate a possible involvement of induced resistance, we focused on the activation of the defense mechanisms of *B. napus* by PdNPs, Pd^2+^ ions and the Pd mix.

**Figure 4.**
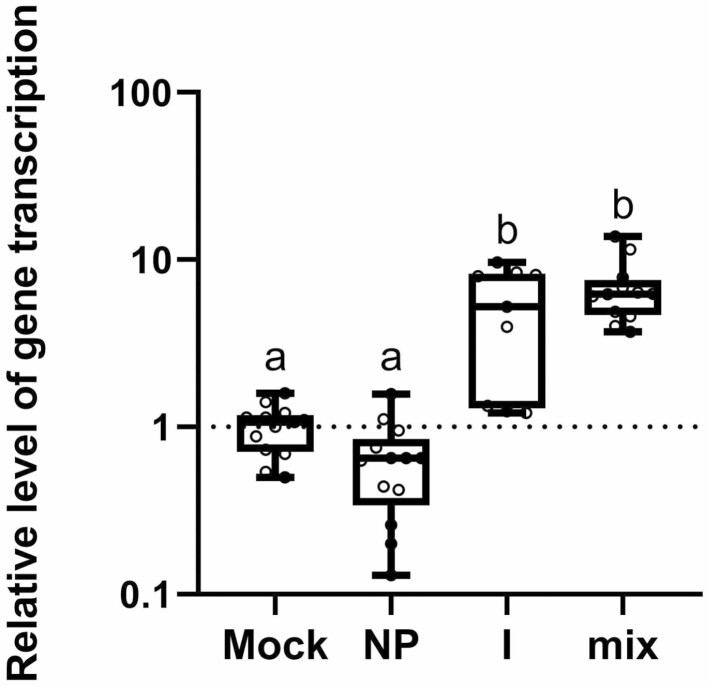
Effect of PdNPs and Pd^2+^ ions on salicylic acid pathway activation. Transcription of *pathogenesis-related 1* (*PR1*) was measured 24 hours after treatment with 35 mg l^–1^ of PdNPs (NP), Pd^2+^ ions (I), or 70 mg l^–1^ of Pd mix (NPs:ions 1:1); *n* = 9, *P* < 0.05 one-way ANOVA.

The activation of plant defence genes was studied in plants treated with a Pd suspensions after 24 hours. This time point presented the situation in cotyledons during inoculation. The major plant defence signalling pathways were investigated. Marker genes of the salicylic acid (SA) pathway were represented by biosynthetic genes *isochorismate synthase 1* (*ICS1*) and *phenylalanine ammonia-lyase 1* (*PAL1*), and an SA-responsive gene the *pathogenesis-related 1* (*PR1*). As other marker genes of the jasmonic acid (JA) pathway, *lipoxygenase 3* (*LOX3*) and *allenoxide synthase* (*AOS*), biosynthetic genes, and the *vegetative storage protein gene* (VSP), a responsive gene, were chosen. As a gene responding simultaneously to the ET and JA pathways; *hevein-like* (*HEL*) was studied. Further genes of interest, *NAC transcription factor RD26* (*RD26*) and *9-cis-epoxycarotenoid dioxygenase* (*NCED3*), were studied as marker genes of abscisic acid (ABA) pathway, where *RD26* is a responsive gene and *NCED3* codes biosynthetic enzyme of ABA. Finally, *senescence-associated gene 12* (*SAG12*) was monitored as a marker of senescence. The results showed that the transcription of *PR1* was significantly increased after Pd^2+^ ion and Pd mix treatment (Fig. 4). Both Pd^2+^ ions and the Pd mix increased gene transcription 6 times compared to the mock control. This indicates activation of the SA pathway in treated plants. PdNPs had no effect on *PR1* transcription change. Other monitored genes had no change in transcription under Pd suspension treatment **[see****Supporting Information—Fig. S1****]**.

### Production of reactive oxygen species and activation of antioxidant enzymes during Pd stress

**Figure 5.**
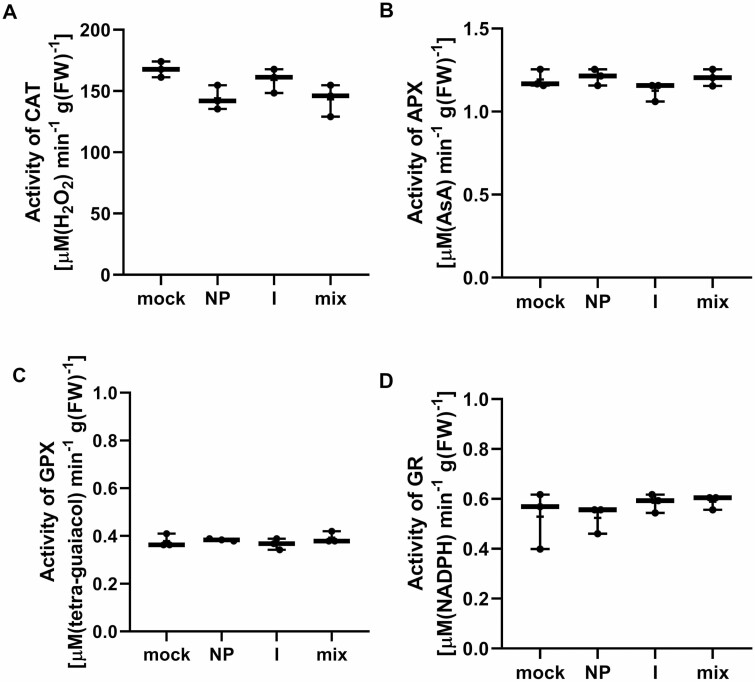
Effect of PdNPs and Pd^2+^ ions on antioxidant enzyme activities. Catalase (A), ascorbate peroxidase (B), guaiacol-dependent peroxidase (C), and glutathione reductase (GR) activity was detected 24 hours after treatment with 35 mg l^–1^ of PdNPs (NP), Pd^2+^ ions (I), or 70 mg l^–1^ of Pd mix (NPs:ions 1:1); *n* = 3, one-way ANOVA.

The production of reactive oxygen species (ROS) and the activation of antioxidant systems are the most common reactions to biotic and abiotic stress in plants. Hydrogen peroxide production was measured in plant tissue 24 h after treatment with PdNPs, Pd^2+^ ions, and Pd mix using DAB staining. Hydrogen peroxide was not detected in *B. napus* cotyledons after the treatment with Pd suspensions. The selected concentration of Pd (35 mg l^–1^ for PdNPs and ions; 70 mg l^–1^ for Pd mix) did not induce the production of hydrogen peroxide (data not shown). Antioxidant enzymes involved in ROS scavenging were also monitored. Catalase, ascorbate peroxidase, guaiacol-dependent peroxidase and glutathione reductase were measured in cotyledons 24 hours after Pd treatment (Fig. 5). None of the Pd suspensions had an effect on the changes in enzyme activity.

### Impact of PdNPs and Pd^2+^ ions on plant fitness

**Figure 6.**
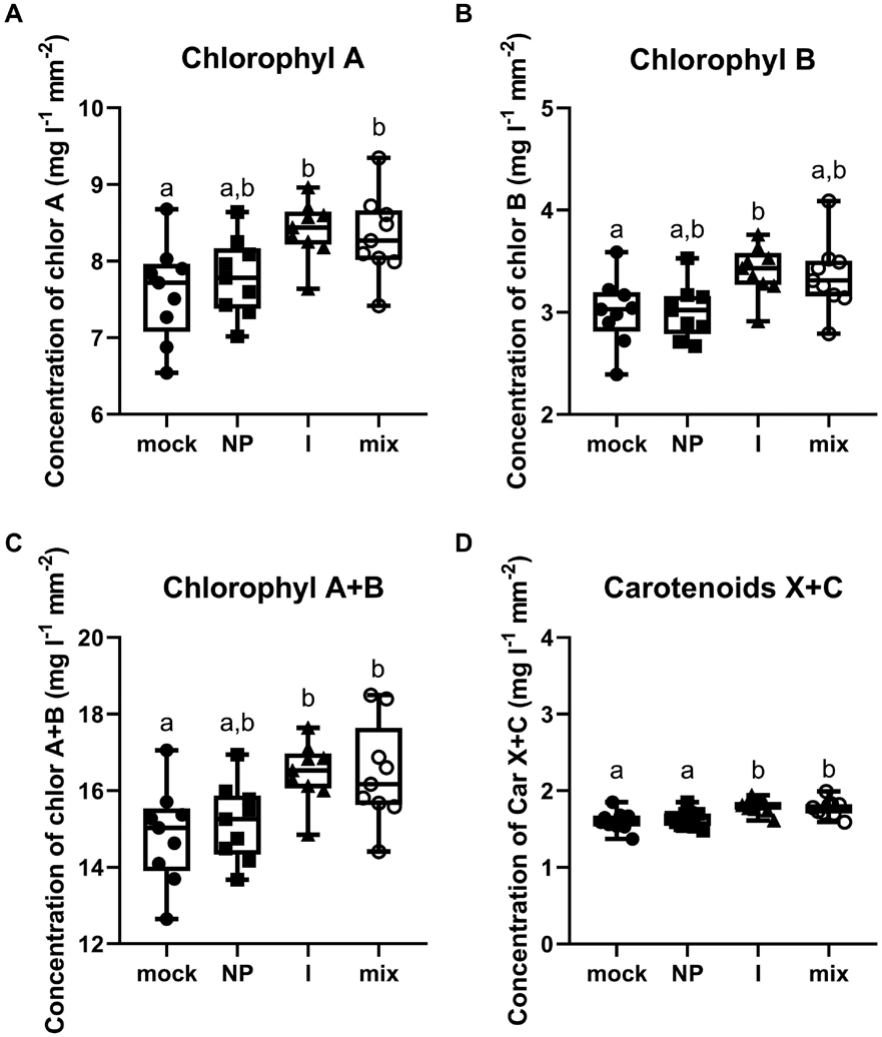
Impact of palladium nanoparticles and Pd^2+^ ions on chlorophyll and carotenoid content. Concentration of chlorophyll *a* (A), *b* (B), *a*+*b* (C), and carotenoids (D) was measured 10 days post-treatment with 35 mg l^–1^ of PdNPs (NP), Pd^2+^ ions (I), or 70 mg l^–1^ of Pd mix (NPs:ions 1:1); *n* = 9, *P* < 0.05.

Exerting energy for defence responses can be visible via changes in leaf vitality. When plants are exposed to some stresses, decreasing plant tissue production can be visible. Leaf areas and dry mass of cotyledons were measured 10 days after infiltration (Table 1). PdNPs and Pd^2+^ ions had no statistically proven effect on the leaf area. No other impacts, such as the yellowing of leaves or lesions caused by the phytotoxic effect of Pd, were observed. The contents of chlorophylls *a* and *b*, as well as carotenoids, were measured after Pd suspensions treatment (Fig. 6). Neither chlorophyll *a* nor *b* was changed in plant leaves after PdNP treatment. In contrast, Pd^2+^ ion treatment increased the content of both chlorophylls. The concentrations of chlorophylls *a* and *b* were increased by 0.88 and 0.5 mg mL^–1^, respectively. The total chlorophyll concentration was increased to 1.63 mg mL^–1^. The results showed that cotyledons infiltrated by Pd^2+^ ions and Pd mix produced more chlorophyll compared with the mock control. The concentration of carotenoids was also measured (Fig. 6). The concentration of carotenoids was significantly increased in the case of plants treated with Pd^2+^ ions and Pd mix. PdNP treatment had no effect on carotenoid content.

**Table 1.** Impact of PdNPs and Pd^2+^ ions on the development of *B. napus* cotyledons. The dry mass and relative area of the cotyledons were measured 10 days after treatment with 35 mg l^–1^ of PdNPs (NP), Pd^2+^ ions (I), or 70 mg l^–1^ of Pd mix (NPs:ions 1:1); *n* = 15-18, one-way ANOVA.

Sample	Concentration of Pd [mg l^–1^]	Form of Pd	Dry mass [%]	Relative leaf area [%]
Mock	0	—	7.59 ± 0.43	100.00 ± 16.57
NP	35	Nanoparticles	7.20 ± 0.43	98.58 ± 21.06
I	35	Ions	7.22 ± 0.51	93.75 ± 15.97
Mix	70	Mixture of nanoparticles and ions (1:1)	7.39 ± 0.49	93.10 ± 18.02

## Discussion

Although PdNPs are being released into the environment in ever-increasing quantities, very little attention has been paid to their effects on living organisms. Several publications have reported on the bioaccumulation of PdNPs in plants and their toxic effects (reviewed in Burketova *et al.*, 2022). The uptake of PdNPs was found in barley (Battke *et al.*, 2008) and *Sinapis alba* (Kinska *et al.*, 2018). The uptake of PdNPs by the roots and their transport within the plants was dependent on the size of the NPs. NPs with a diameter of 1–15 nm were found to accumulate to a significantly higher extent in leaves and reduce leaf length compared to Pd particles with a size of 1 µm (Battke *et al.*, 2008). The uptake of metal NPs by roots and their transport within the plant depend on the chemical properties of the NPs. Some of them, including PdNPs, show chemical reactivity in the natural environment, especially at lower pH values and compounds that are exudated by the roots into soil and can cause the release of free metal ions. Given the ability of plants to accumulate metals and biosynthesise NPs (Vishnukumar *et al.*, 2017), it is suspected that NPs enter the plant either directly in the form of NPs or as ions previously released from NPs in the soil and subsequently converted to NPs by the plant (Battke *et al.*, 2008). In our experiments, the PdNPs were artificially infiltrated into leaf apoplast. We do not have sufficient data to hypothesise, whether the fate of PdNPs in apoplastic space was similar, but considering the flow of the apoplastic fluid rich in ions, proteins and other compounds, we cannot exclude a similar release of Pd^2+^ ions.

In addition to plants, PdNPs also have a harmful effect on microorganisms. Their antimicrobial activity has been demonstrated mainly on animal/human pathogens. Data on plant pathogens are scarce (reviewed by Burketova *et al.*, 2022). Osonga *et al.* (2020) reported a size-dependent fungistatic effect of PdNPs on *F. oxysporum* and *C. gloeosporoides*. Our study shows that the suspension of PdNPs inhibited the germination of *P. lingam*, but the effect was due to Pd^2+^ ions and not to the NPs alone (Fig. 2), and this inhibition was not found, when the PdNPs were applied on the grown mycelium. Thus, we expand current knowledge about microorganisms that are sensitive to Pd.

Considering the role of NPs or metal ions in the interaction between plants and microbes, it is worth noting that both forms of metals can also affect plant physiology and subsequently lead to plant resistance or susceptibility to pathogens and pests. These contradictory effects are based on hormesis, which manifests itself in the compound having a beneficial effect at low doses and a harmful effect at high doses (reviewed by Morkunas *et al.*, 2018). Similar to pathogens, some heavy metals are able to activate defence responses such as the production of reactive oxygen species (ROS), antioxidant enzymes, pathogenesis-related proteins and phytohormones, indicating a convergence between biotic and abiotic stresses. Plant resistance triggered by prior inoculation with a pathogen or treatment with a potent compound is termed as ‘induced resistance’ (Kuc, 2001; Oostendorp *et al.*, 2001). A wide range of compounds including heavy metals showed this activity (Tamás *et al.*, 1997; Utriainen *et al.*, 1998; Walters *et al.*, 2013; Burketova *et al.*, 2015; Morkunas *et al.*, 2018; Zhou and Wang, 2018; Devi *et al.*, 2020). The aim of our work was to extend the current knowledge on the potential toxicity of PdNPs to the economically important crop oilseed rape (*B. napus*) and its fungal pathogen *P. lingam*. In the pathosystem studied, induced resistance of *B. napus* to *P. lingam* has also been reported (Liu *et al.*, 2006; Sasek *et al.*, 2012b; Novakova *et al.*, 2016). The main signalling molecule involved was salicylic acid. In our study, we wanted to confirm or refuse our hypothesis, that PdNPs have impact on plant fitness and defence system, and thereby influence the resistance of *B. napus* to *P. lingam*.

We pretreated *B. napus* plants with a suspension of PdNPs and monitored whether the extent of symptoms induced after subsequent inoculation with *P. lingam* was affected. Since there are several studies showing that metal NPs can release metal ions into the solution or that these ions may be present in residual concentrations in the solution after synthesis of NPs (Palencia *et al.*, 2014), we used palladium chloride solution as a control treatment at the increasing concentrations. As can be seen in Fig. 3A, there was indeed a reduction in the extent of symptoms after the treatment with palladium, starting at a concentration of 35 mg l^–1^. However, the results show that the reduction of *P. lingam* symptoms in *B. napus* cotyledons was caused by the Pd^2+^ ions. These results thus suggest that palladium ions are primarily responsible for reducing the magnitude of *P. lingam*-induced symptoms and that PdNPs do not contribute to this effect.

These experiments were complemented by monitoring the direct antifungal effect of palladium *in vitro* (Fig. 2). From these results, it is clear that palladium mainly affects *P. lingam* spore germination, because if palladium was added 72 hours after the spores started to germinate and the mycelium appeared, no suppression of the growth *in vitro* was observed. The results show that, again in this case, Pd ions are mainly responsible for the antifungal effect and the PdNPs alone do not contribute to the effect. This result pointed out antimicrobial activity of Pd ions as probable mechanism of *P. lingam* lesions reduction. On the other hand, it is difficult to determine unequivocally whether the reduction in symptoms was caused solely by the palladium antimicrobial activity or whether the induced resistance mechanisms were also involved. Typical resistance inducers do not have an antimicrobial activity; however, this cannot be excluded, as some fungicides have the ability to induce plant resistance in addition to direct antifungal activity (Herms *et al.*, 2002; Schillheim *et al.*, 2018), and in addition, heavy metals have been previously reported to induce resistance to pathogens in plants (Kuc, 2001). To determine whether induced resistance indeed plays a role, we performed an experiment in which PdNPs and Pd^2+^ ions were applied 24 hours after the inoculation of plants with the pathogen. However, in this case, there was no reduction in symptom development (Fig. 3B). Thus, we hypothesized that induced resistance may be also involved since a direct antifungal effect *in planta* is unprobable because of the lack of effect in Pd post-inoculation treatment of plants. As we showed in one of the previous studies (Sasek *et al.*, 2012b), the spores of *P. lingam* germinated only 48 hours after inoculation, thus 24 hours after inoculation in this experiment the Pd treatment may affect still spores present in mesophyll and not a developed mycelium.

To investigate whether palladium is capable of inducing resistance in plants, we monitored the transcription of marker genes normally involved in induced resistance. We found that palladium ions activate the expression of the *PR1* gene, which is generally expressed during SA-dependent responses to biotic stress and has been implicated in induced resistance (Walters *et al.*, 2013; Burketova *et al.*, 2015). Moreover, our previous research showed that both specific and induced resistance in *B. napus* to *P. lingam* is regulated by SA (Sasek *et al.*, 2012b; Kim *et al.*, 2013; Novakova *et al.*, 2016). In contradiction, the transcription of genes involved in SA biosynthesis *ICS* and/or *PAL* was not detected 24 hours post inoculation. This discrepancy could be explained by possibly transient transcription of these genes, which could occur earlierly.

Since the common response of plants to NPs or heavy metal treatment is the formation of reactive oxygen species in the tissues, as has been described for silver NPs (Jiang *et al.*, 2014; Hu and Xianyu, 2021), we monitored the presence of hydrogen peroxide in the tissues of cotyledons using an *in situ* DAB staining method, while also monitoring the activities of antioxidant enzymes. Our results show that neither PdNPs nor ions induce oxidative stress in *B. napus*. At the same time, we also did not detect toxicity at the level of chlorophyll content in palladium-treated cotyledons, which surprisingly showed an increase in chlorophyll content (Fig. 5). In the case of Pd^2+^ ion treatment and the mix (PdNPs and Pd^2+^ ions) treatment, this increase is not easy to explain, but we hypothesize that this could occur as in the case of heavy metal treatment of plants, where it has been described that cadmium ions can substitute the magnesium atom in the tetrapyrrole ring, thus inhibiting chlorophyll function at the level of photosystem 1, i.e., reducing chlorophyll fluorescence and thus the efficiency of photosynthesis (Bertrand and Poirier, 2005; Bechaieb *et al.*, 2016). Therefore, it is possible that the plant may then tend to compensate for this reduction in photosynthetic activity by higher chlorophyll synthesis. Finally, we did not detect the phytotoxic activity of PdNPs and ions even on the basis of growth parameters, where leaf area and plant weight were evaluated (Table 1).

The fate of palladium ions and chiefly NPs in plant tissues is another question, which should be discussed, because plants have the ability to detoxify metals by transforming them into NPs (Beattie and Haverkamp, 2011; Marchiol *et al.*, 2014). This phenomenon cannot be ruled out in the case of palladium ions and *B. napus*, given that *B. napus* has been described as a hyperaccumulator of NPs and is used in the remediation of contaminated soils (Purakayastha *et al.*, 2008; Ferreyroa *et al.*, 2017; Rosca *et al.*, 2021). In summary, in our experiments we have demonstrated the inhibition of spores germination of a PdNP suspension against *P. lingam* both *in vitro* and *in planta* and we showed that the palladium ions contained in the nanoparticle suspension are responsible for this effect. We could not find any phytotoxic effect of either PdNPs or ions on the cotyledons of *B. napus*. However, palladium ions in higher concentrations stimulated plant defence responses regulated by salicylic acid. Therefore, we cannot exclude that induced resistance was also involved in the decrease of *P. lingam* symptoms on cotyledons. Based on these results, we assume that PdNPs do not pose a major risk to *B. napus* in terms of direct exposure. However, due to the pronounced antimicrobial activity of palladium ions, as PdNPs tend to release ions depending on the solution/capping agent (Gioria *et al.*, 2020), it cannot be excluded that they may negatively affect the soil microbiome and thus indirectly have a negative impact on plant productivity. More attention should be paid to this aspect of PdNPs in future research.

## Supporting Information

The following additional information is available in the online version of this article—

**Figure S1.** Relative gene transcription of other markers 24 hours after exposure of PdNPs and Pd^2+^.

**Table S1.** List of primers used for gene transcription analysis.

**Experimental data.** Detailed data of experiments.

plad004_suppl_Supplementary_Material

plad004_suppl_Supplementary_Data

## Data Availability

All experimental data are provided in the Supplementary Information.
