# Supplementary material for: Impact of palladium nanoparticles on plant and its fungal pathogen. A case study: Brassica napus–Plenodomus lingam
Source: AoB Plants. 2023 Feb 2;15(2):plad004. doi: 10.1093/aobpla/plad004 (PMC10037078; doi:10.1093/aobpla/plad004)
Supplement: plad004_suppl_Supplementary_Material [file plad004_suppl_supplementary_material.pdf]

Supporting information

Fig S1.: Effect of PdNPs and Pd^{2+} ions on defence pathways (salicylic acid, jasmonic acid, ethylene), senescence and abscisic acid pathway activation. Transcriptions of marker genes were measured 24 h post treatment with 35 mg l^{-1} of PdNPs (NP), Pd^{2+} ions (I) or 70 mg l^{-1} of Pd mix (NPs:ions 1:1)

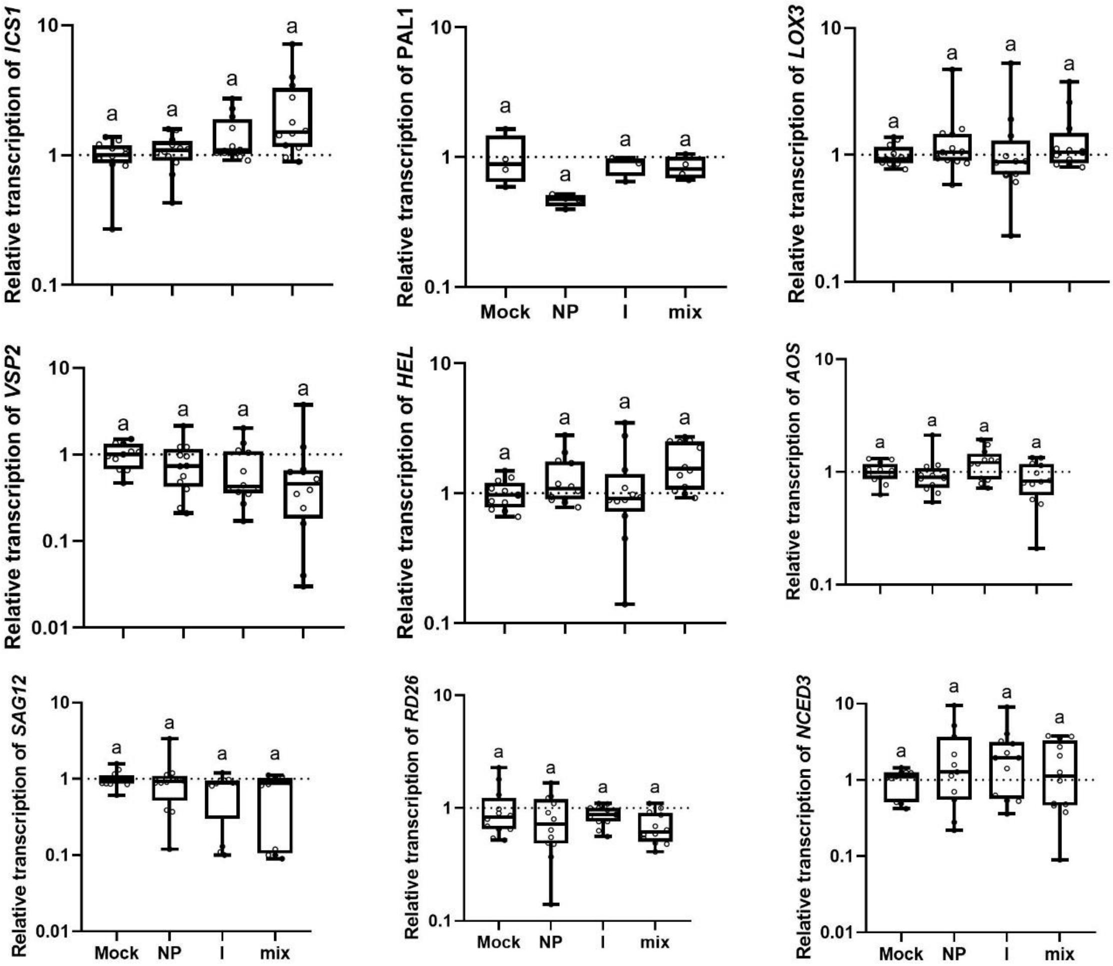

Table S1: List of primers

Gene	EST	Name	Sequence
**ACT**	AF111812	F	CTGGAATTGCTGACCGTATGAG
		R	TGTTGGAAAGTGCTGAGGGA
**PR1**	BNU21849	F	CATCCCTCGAAAGCTCAAGAC
		R	CCACTGCACGGGACCTAC
**ICS1**	EV225528	F	CAAACTCATCATCTTCCCTC
		R	AGCGTGACTTACTAACCAG
**PAL**	DQ341308.1	F	GACTAATCTCATCTCGCAAG
		R	ATTCTCCTCCAAGTGTCTTAG
**LOX3**	EV113862	F	GAAGTTTATGGCGGTGGT
		R	CCTGTTTCTACGGTTAGGA
**VSP2**	CN726858	F	CCTCTCACTTTCACTTCTCTTGC
		R	GTTCGGCTTCGTCCTCAATG
**AOS**	EV124323	F	CGCCACCAAAACAACAAAG
		R	GGGAGGAAGGAGAGAGGTTG
**HEL**	FG577475	F	GGAACACAAGGACTAATGC
		R	TTTCGATAGCCATCACCA
**SAG12**	EV109649	F	TATAGAAGGAGCAACGCA
		R	AAACGCAGTATCCATTAGAC
**RD26**	GT085050.1	F	ATCGGTCTTTCAATCTTCCT
		R	GAGTTCATCTGCAAATTCCT
**NCED3**	EV137674	F	CGATTTGCCTTACCAAGTCAG
		R	TTTATCCCTTCCGGTGAGAA
